# Use of levosimendan in hemodynamic management of heart failure in two neonates with intracranial arteriovenous shunts: a case series

**DOI:** 10.1186/s13052-023-01537-1

**Published:** 2023-10-15

**Authors:** Francesca Landolfo, Paola Giliberti, Domenico Umberto De Rose, Flaminia Pugnaloni, Alessandra Santisi, Claudia Columbo, Ludovica Martini, Maria Paola Ronchetti, Luca Di Chiara, Alessandra Toscano, Carlo Gandolfo, Andrea Dotta, Irma Capolupo

**Affiliations:** 1 Neonatal Intensive Care Unit, “Bambino Gesù” Children’s Hospital IRCCS, Rome, 00165 Italy; 2 Pediatric Cardiac Intensive Care Unit, “Bambino Gesù” Children’s Hospital IRCCS, Rome, 00165 Italy; 3 Perinatal Cardiology Unit, “Bambino Gesù” Children’s Hospital IRCCS, Rome, 00165 Italy; 4 Neuroradiology Unit, “Bambino Gesù” Children’s Hospital IRCCS, Rome, 00165 Italy

**Keywords:** PRAM, NIRS, Neonate, NICU, Galen vein malformation, Cerebral arteriovenous malformations, Case report

## Abstract

**Background:**

The hemodynamic status of newborns with intracranial arteriovenous shunts (AVSs) may be extremely complex. Mini-invasive hemodynamic monitoring through innovative techniques such as Near-Infrared Spectroscopy (NIRS) and Pressure Recording Analytical Method (PRAM) may help in understanding hemodynamics in newborns with AVSs. Levosimendan is a calcium sensitizer and inodilator, and it is known to improve ventricular function, but its use in newborns is limited. In our cases, we evaluated the effect of levosimendan on hemodynamics through NIRS and PRAM.

**Case presentation:**

Herein, we report the cases of two neonates with intracranial arteriovenous shunts, in whom we used levosimendan to manage cardiac failure refractory to conventional treatment. Levosimendan was used at a dosage of 0.1 mcg/kg/min for 72 h. Combined use of NIRS and PRAM helped in real-time monitoring of hemodynamic effects; in particular, levosimendan determined significant improvement in myocardium contractility as well as a reduction of heart rate.

**Conclusion:**

In two neonatal cases of AVSs, levosimendan led to an overall hemodynamic stabilization, documented by the combination of NIRS and PRAM. Our results suggest introducing levosimendan as a second-line treatment in cases of severe cardiac dysfunction due to AVSs without improvement using standard treatment strategies. Future prospective and larger studies are highly warranted.

**Supplementary Information:**

The online version contains supplementary material available at 10.1186/s13052-023-01537-1.

## Background

Intracranial arteriovenous shunts (AVSs) are rare cerebral defects, including pial arteriovenous malformations (AVMs), vein of Galen malformations (VGAMs), and dural arteriovenous fistulae (DAVF) [1]. High mortality and morbidity rates are still linked to intracranial AVSs in neonates, frequently presenting with high-output cardiac failure [2].

Levosimendan may be ideally suited to provide inotropic support in situations of acute heart failure since it is a calcium sensitizer and inodilator that increases cardiac contractility without raising myocardial oxygen demand or escalating ischemia [3].

We report the cases of two neonates with intracranial AVSs, in whom we used levosimendan to improve hemodynamics. The combination of Near-Infrared Spectroscopy (NIRS) and Pressure Recording Analytical Method (PRAM) demonstrated changes in hemodynamic monitoring in these neonates before and after 72 h of levosimendan administration. When heart failure caused by a vascular steal and consequent cardiac overflow is unresponsive to traditional management, this drug can improve ventricular contractility, decreasing heart rate.

## Case presentation

Medical records of patients with intracranial AVSs admitted to our Neonatal Intensive Care Unit (NICU) in the last two years (January 2020 - July 2022) were revised. Medical history and clinical data were collected for neonates treated with levosimendan, who were included in this case series.

A standard arterial catheter was inserted into an artery (3.5 Fr umbilical artery, 2 Fr radial or femoral artery), and it was connected to MostCare-Up® (Vygon, Vytech, Padua, Italy) hemodynamic monitoring system through a standard pressure transducer. MostCare-Up® is powered by the pressure recording analytical method (PRAM) that analyses arterial waveform, and beat-by-beat data are displayed on a dedicated monitor with a frequency sampling of 1000 Hz with a high degree of precision [4]. Real-time monitoring of several parameters permitted to detect beat-by-beat information about cardiac preload, afterload, contractility, and tissue oxygen delivery, allowing to the choice of the most appropriate drug treatment and monitoring hemodynamics changes in response to therapeutic strategies.

A near-infrared spectrometer (Invos 5100; Somanetics Corp, Troy, Michigan) equipped with two independent emittent-sensor pairs was used for simultaneous measurement of regional cerebral and splanchnic tissue oxygen saturation (rSO2c and rSO2s, respectively), calculating fractional tissue oxygen extraction (FTOE) that reflects the balance between oxygen supply and oxygen consumption [5].

Brain magnetic resonance imaging (MRI) exams were performed on a 3 T scanner (MAGNETOM Skyra, Siemens, Erlangen, Germany).

Data are presented as numbers and percentages for categorical variables. Continuous variables are expressed as means ± standard deviation if they were normally distributed or as the median and interquartile range (IQR) if normality could not be accepted, according to the D’Agostino-Pearson test. The distribution of data was evaluated using the program MedCalc (version 12.7 for Windows).

Of 11 patients with intracranial AVSs, admitted to our NICU during the study period, two infants received levosimendan infusion and were included in this case series. Clinical data are reported in Table 1.

**Table 1:** Clinical details of included patients

	*Patient 1*	*Patient 2*
Sex	Female	Male
Birthweight (grams)	2000	3690
Birthweight Z-score (SD)	0.47	0.99
Gestational age (weeks + days)	33 + 2	39 + 0
Maternal age (years)	37	36
In vitro fertilization	No	No
Prenatal heart failure	Yes	No
Prenatal diagnosis	Yes	No
Intubation at birth	Yes	No
Apgar score at 1st minute	5	8
Apgar score at 5 min	8	9
Admission pH	7.14	7.32
Admission lactates (mmol/l)	2.5	2.8
Admission Bicêtre score	2	2
Maximum NT-proBNP (pg/ml)	129 077	265 736
Maximum vasoactive inotropic score	6	9.7
Number of embolizations	1	2
In-hospital mortality	Yes	No

### Patient 1

**Fig. 1:**
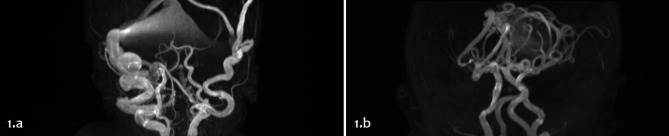
Brain MRI findings of intracranial AVSs in sagittal planes, revealing a DAVF of the posterior cranial fossa in patient 1 (**1.a**) and a VGAM in patient 2 (**1.b**)

**Fig. 2:**
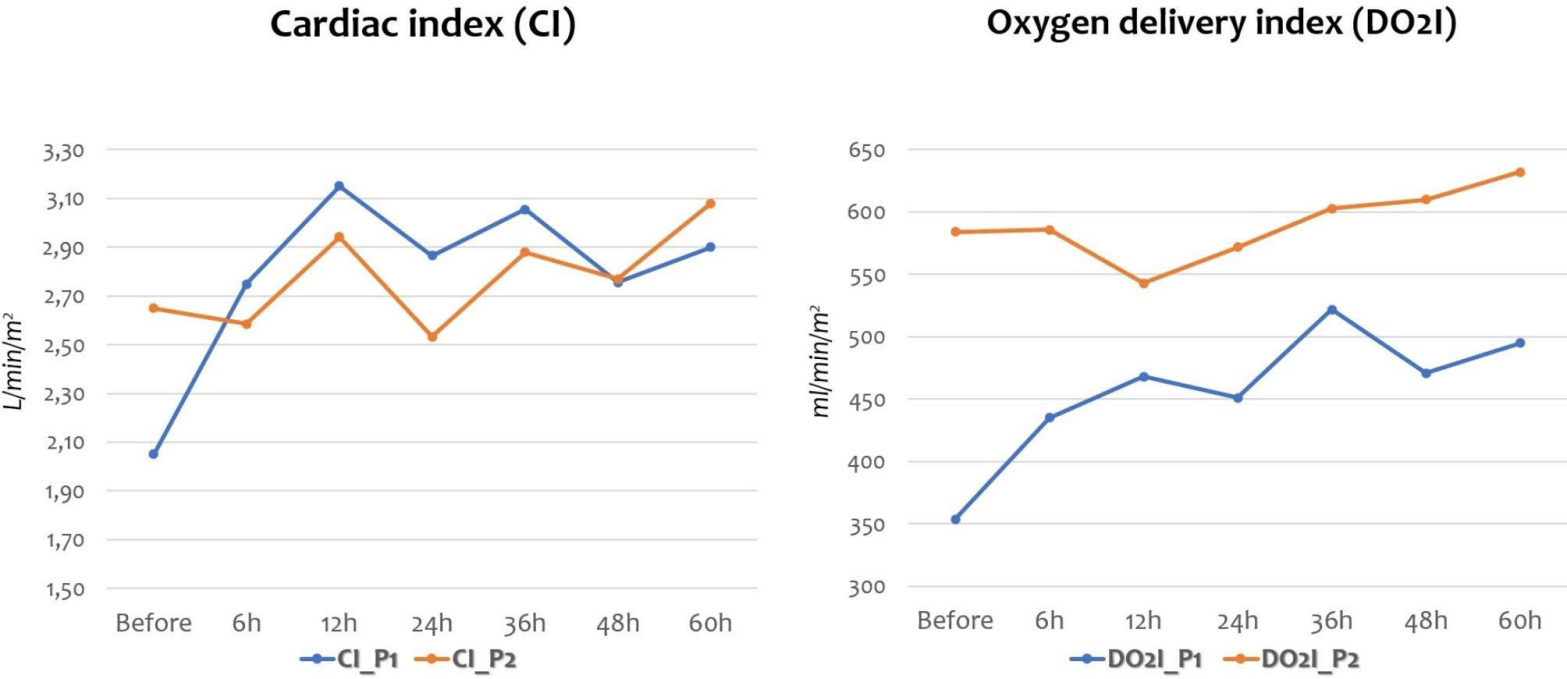
Improvement in cardiac index (CI) and oxygen delivery index (DO2I) in patient 1 (P1) and patient 2 (P2) before and after Levosimendan administration

Patient 1 (P1) had a prenatal diagnosis of suspected VGAM: she was born preterm (33 weeks) via cesarean section because of pathological umbilical artery Doppler parameters and fetal heart failure with right heart enlargement. She needed mechanical ventilation since birth because of respiratory distress. Brain MRI revealed a dural sinus malformation (DSM) of the posterior cranial fossa with venous congestion (Fig. 1.a). She required continuous infusion of diuretics (etacrynic acid, up to 0.2 mg/kg/h) and milrinone (0.75 mcg/kg/min) to improve her hemodynamics; given the still unstable conditions, levosimendan (0.1 mcg/kg/min) was used for 72 h (stopping milrinone) with a transient significant improvement of hemodynamic parameters at MostCare-Up® monitoring (Table 2): cardiac index and oxygen delivery index progressively improved (Fig. 2 – P1). She underwent a first embolization of the AVS at 5 days of age. At 8 days of age, she presented with pulmonary haemorrhage related to pulmonary oedema and gradual multiorgan failure with renal failure and coagulation abnormalities, for which required serial frozen plasma transfusions, antithrombin, and vitamin K. A second embolization procedure was hypothesized, but it was not possible due to clinical instability. She died after 19 days of life.

**Table 2:** Changes in hemodynamic parameters in patient 1

*Patient 1*	*Before levosimendan*	*After 48 h since levosimendan infusion*
Systolic pressure	52.0 (49.0–54.0)	64 (60.0–66.0)
Diastolic pressure	25.0 (24.0–27.0)	38.0 (34.0–44.0)
Dicrotic pressure	24.0 (23.0–26.0)	30.0 (28.0–31.0)
Pulse rate	155 (148–159)	154 (135–158)
CI	2.05 (1.94–2.29)	2.90 (2.53–3.04)
DO2I	354 (334–403)	495 (431–518)
Cerebral FTOE	0.48 (0.41–0.53)	0.51 (0.45–0.56)
Renal FTOE	0.31 (0.22–0.46)	0.62 (0.49–0.72)

CI: cardiac index; DO2I: oxygen diffusion index; FTOE: fractional tissue oxygen extraction

### Patient 2

Patient 2 (P2) received no prenatal diagnosis and was born in a 1st level hospital. He was transferred to our NICU because of suspected congenital heart disease, given the finding of a systolic murmur and desaturations at birth. Their first hypothesis was a total anomalous pulmonary venous return (TAPVR) and was referred while on prostaglandin infusion. At NICU admission, echocardiography and cranial ultrasound allowed us to diagnose a high-output heart failure and a muscular ventricular septal defect (VSD), in the context of a VGAM (choroidal type) confirmed by brain MRI (Fig. 1.b). Prostaglandin infusion was stopped, and heart failure was managed with continuous infusion of diuretics (etacrynic acid, up to 0.2 mg/kg/h) and milrinone (0.75 mcg/kg/min). He presented with mild pulmonary haemorrhage, which improved with high-frequency oscillatory ventilation and frozen plasma transfusions. Similarly to P1, levosimendan infusion (0.1 mcg/kg/min) for 72 h allowed us to improve clinical stability and hemodynamic parameters at MostCare-Up® monitoring (Table 3): cardiac index and oxygen delivery index progressively improved (Fig. 2 – P2). He underwent two embolization procedures of the VGAM at 10 and at 18 days of age respectively. The postoperative course was uneventful, with extubation at 35 days of life. He required in total 35 days of mechanical ventilation and further 26 days of non-invasive respiratory support. The infant had transient difficulties in enteral feedings due to chronic tachypnea and this prolonged hospital stay. The infant was discharged home at 3 months of life.

**Table 3:** Changes in hemodynamic parameters in patient 2

*Patient 2*	*Before levosimendan*	*After 48 h since levosimendan infusion*
Systolic pressure	87 (83–92)	96 (92–99)
Diastolic pressure	46 (45–47)	43 (41–45)
Dicrotic pressure	48 (46–54)	59 (57–60)
Pulse rate	158 (143–166)	152 (145–154)
CI	2.65 (2.56–2.76)	3.05 (2.98–3.18)
DO2I	584 (375–606)	632 (535–690)
Cerebral FTOE	0.42 (0.40–0.44)	0.32 (0.32–0.32)
Renal FTOE	0.43 (0.41–0.45)	0.50 (0.47–0.52)

CI: cardiac index; DO2I: oxygen diffusion index; FTOE: fractional tissue oxygen extraction

## Discussion and conclusions

In this manuscript, we provided for the first time a detailed description of hemodynamic changes after the use of levosimendan in neonates with intracranial AVSs. Although transiently, it allowed us to transfer both our patients to the operating theatre for embolization. Embolization by endovascular treatment has become the standard treatment for cerebral arteriovenous shunts, in particular for VGAM [2]. When possible, the procedure should be postponed after 3 months of age, considering the worse outcomes reported in infants weighing less than 5 kg, including adverse cardiac effects and major cerebral complications due to rebleeding [6].

**Fig. 3:**
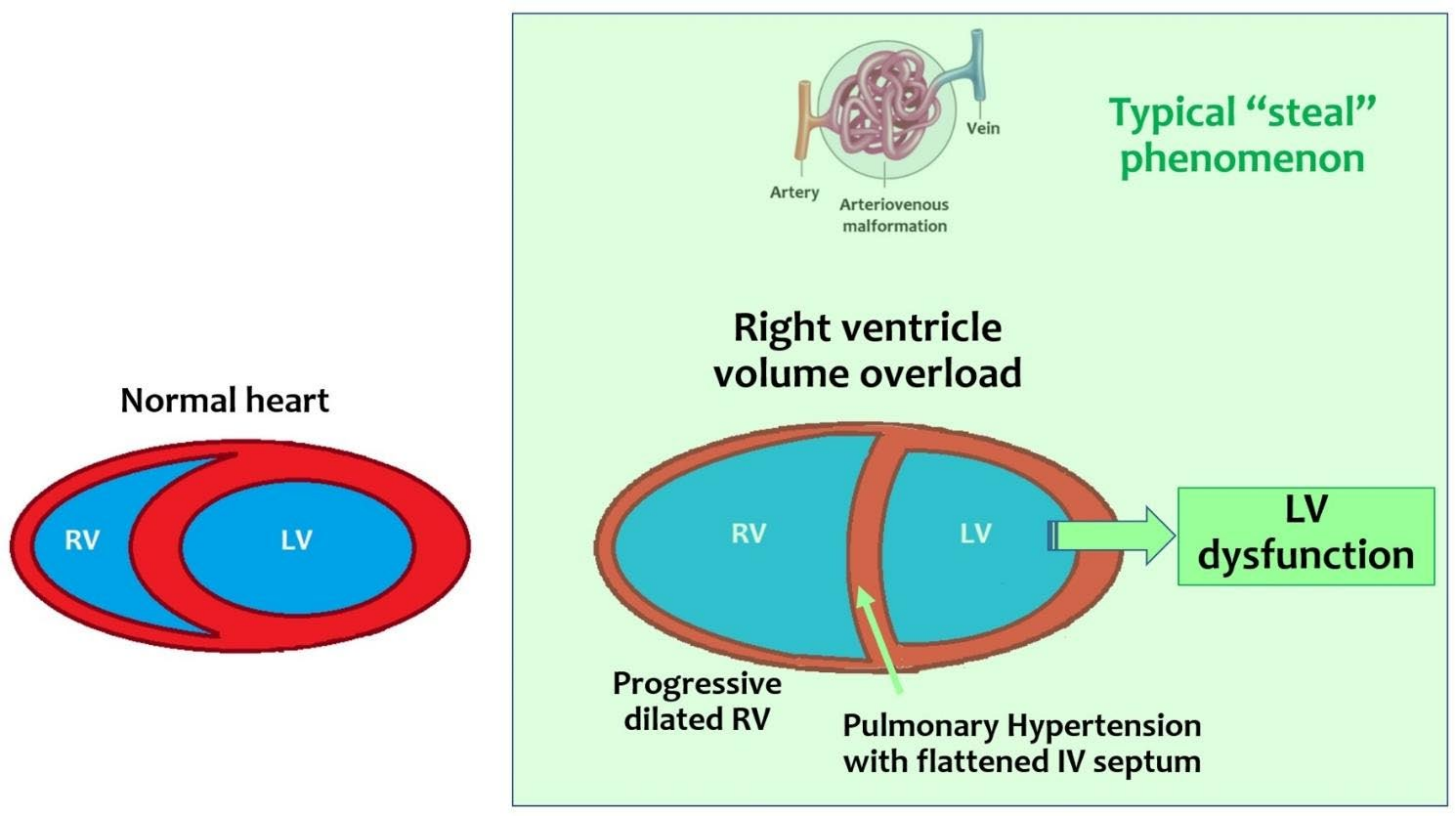
Cardiac hemodynamic findings and physiological considerations in intracranial AVSs

However, high-output cardiac heart failure is the most common presentation of intracranial AVSs in the neonatal period (Fig. 3) [2], with features overlapping to those of congenital heart disorders that characterize the clinical picture: cyanosis, compromised peripheral pulses, and a cardiac murmur (often audible also through the fontanelle) [7, 8]. Intracranial AVSs often determine high-output heart failure characterized by increased venous return and increased afterload to the right ventricle (RV), resulting in a distended and non-compliant RV and increased pulmonary blood flow with pulmonary hypertension. The left ventricle (LV) often has normal or hyperdynamic function; however, the cardiac output (CO) is unable to meet the metabolic demand of the systemic organs resulting in lactic acidosis [9]. The prognosis is much worse than in those presenting later in childhood, with a higher risk of death due to cardiac failure [10]. Furthermore, survivors with previous heart failure had significantly worse neurodevelopmental outcomes [2].

On the other hand, these infants present with severe unstable conditions and require ventilatory support, inotropes and diuretics to improve hemodynamics, thus allowing them to undergo an embolization procedure.

The main aim of medical treatment of AVS-related hemodynamic changes is represented by ensuring blood flow to non-cerebral circulation through decreasing pulmonary overflow, reducing cardiac preload, and improving myocardial function (Table 4).

**Table 4:** Role of each intervention in hemodynamic management of neonatal intracranial AVSs with heart failure

*Intervention*	*Drug doses*	*Aim*
Sedation and mechanical ventilation	Fentanyl 0.5-2.0 μg/kg/h iv Remifentanil 0.1-1.0 μg/kg/min iv	To optimize cardiopulmonary interactions
Inotropic or vasopressor agents	Epinephrine 0.1-1 μg/kg/min iv Dobutamine 5–10 μg/kg/min iv Dopamine 5–10 μg/kg/min iv Vasopressin 0.0001–0.001 μg/kg/min iv	To improve cardiac contractility and increase systolic pressure
Diuretics	Ethacrynic acid 0.1–0.2 mg/kg/h iv	To reduce volume overload
Milrinone	Milrinone 0.3–0.75 μg/kg/min iv	To improve myocardial LV and RV performance and reduce pulmonary hypertension
Pulmonary vasodilators	Inhaled nitric oxide (iNO) 5–20 ppm Sildenafil 1.6 mg/kg/day iv	To reduce pulmonary vascular resistance and RV afterload
Prostaglandins infusion	PGE1 0.01 μg/kg/min iv	To maintain patent ductus arteriosus directing the increased blood flow through the pulmonary arteries to the systemic circulation and reduce pulmonary arterial pressure
Levosimendan	72 h continuous iv infusion of 0.1 μg/kg/min	To improve myocardial LV performance without increasing oxygen demand

Diuretics and adequate fluid balance management are essential to reduce cardiac preload. Inotropic and vasopressor agents can support ventricular dysfunction (especially low-dose epinephrine and dobutamine), but they should be cautiously used to avoid excessive systemic vasoconstriction and to increase cardiac oxygen consumption.

The use of direct pulmonary vasodilators therapy (i.e. inhaled Nitric oxide - iNO) has been reported [11, 12] in order to reduce pulmonary vascular resistance (PVR). On the contrary, in the setting of pulmonary venous hypertension, the literature suggests that the use of pulmonary vasodilators to decrease PVR can lead to clinical deterioration [13] due to a further increase in pulmonary flow with the potential for lung edema or hemorrhage.

Prostaglandin infusion may be useful in patients with intracranial AVSs, presenting with refractory heart failure and suprasystemic pulmonary hypertension [14], to decompress right-to-left ductal shunting and thereby maintain an adequate systemic blood flow while awaiting definitive treatment. However, given the typical pulmonary venous hypertension of these infants and their pulmonary hemorrhages, we did not consider the use of pulmonary vasodilators or prostaglandin infusion.

Cardiopulmonary dysfunction could also be managed with inodilators such as milrinone, although it can cause an increase in heart rate and a decrease in blood pressure [15]. Intravenous milrinone has a pulmonary vasodilator effect but significantly improves myocardial performance: however, literature still lacks *in-vivo* measurements of cardiac output and systemic vascular resistance (SVR) after milrinone administration in newborn animal models with pulmonary hypertension due to the difficulty of performing double thoracotomies [16].

Despite the use of milrinone, both our patients persisted in having clinically relevant pulmonary hypertension (PH) and required the administration of levosimendan in order to stabilize them and allow the transport to the operating theatre.

De Rosa et al. previously reported the use of levosimendan in two of 12 neonates with VGAM who underwent endovascular embolization: both survived, whereas all-cause mortality of 58.3% (7/12) was reported in this cohort [17]. However, the aim of their paper was to describe the global outcome of their neonates with VGAM, and full details of levosimendan use were not reported. Conversely, herein we documented changes in hemodynamics parameters after levosimendan infusion at beat-by-beat analysis through PRAM with better cardiac output.

Levosimendan is a new inotropic agent belonging to calcium sensitizers, that improve myocardial contractility by increasing the sensitivity of troponin C for calcium, without increasing myocardial oxygen consumption, like the other inotropes [18]. First studies on levosimendan use are related to neonates with congenital heart disorders and congenital diaphragmatic hernia [19, 20]. Levosimendan can be used alone or in combination with other inotropic or vasopressor medications; however, given the risk of hypotension due to vasodilation by opening ATP-dependent potassium channels, careful monitoring is required [9].

The introduction of levosimendan in our two patients constituted the “bridge” to endovascular treatment of intracranial AVSs, whereas milrinone was not sufficient. These data are in line with Lechner’s findings, who previously studied the effects of levosimendan and milrinone in infants with congenital heart disorders: they observed an increase in the cardiac index in the levosimendan group, whereas cardiac index remained stable in the milrinone group [21]. In our two neonates, the use of PRAM monitoring allowed us to demonstrate significantly improved hemodynamic parameters, with a higher cardiac index (CI) after levosimendan infusion. The main hemodynamic parameters can also be obtained using other non-invasive devices, such as the cardiac index using echocardiography and the oxygen delivery index (DO2I) using Ultrasonic Cardiac Output Monitor (USCOM): however, we found that the main advantage of PRAM monitoring is the availability of a beat-by-beat analysis for all clinicians who take care of these neonates, especially those who are not able to perform functional echocardiography, and without stressing these fragile infants with evaluations with multiple devices.

Conversely, our findings warn of the limitations of NIRS monitoring in detecting changes during regional monitoring in these patients: we observed controversial results about cerebral FTOE before and after levosimendan infusion. We speculated that the presence of AVSs influences cerebral fractional tissue oxygen extraction, but the sole introduction of a drug could not be sufficient to compensate for the continuous typical “steal” phenomenon of these anomalies. In most severe cases, only the endovascular treatment can minimize flow shunt and significantly modify regional oxygen extraction. Furthermore, NIRS trends over time are more important than single values and can inform about hemodynamic instability.

To our knowledge, this is the first case series investigating in detail the changes in hemodynamics in levosimendan-treated neonates with intracranial AVSs.

Neonates with intracranial AVSs require multidisciplinary care, from preoperative stabilization to endovascular embolization, and pharmacological treatment should be discussed with different involved specialists [9]. The use of levosimendan could improve hemodynamics when conditions under conventional drugs are too unstable to allow neonates to undergo endovascular procedures. Multicenter randomized studies would need to provide evidence about its utility, but the rarity of intracranial Avs limits this type of research. Considering these disorders are life-threatening, even the use of off-label drugs in neonatal age should be promoted. Therefore, our experience could be a starting point for discussing the role of levosimendan in these congenital malformations.

### Electronic supplementary material

Below is the link to the electronic supplementary material.

Supplementary Material 1

## Data Availability

All data generated or analysed during this study are included in this published article.

## List of abbreviations

AVM: Arteriovenous malformation
AVSs: Arteriovenous shunt
DAVF: Dural arteriovenous fistula
DO2I: Oxygen delivery index
DSM: Dural sinus malformation
FTOE: Fractional tissue oxygen extraction
iNO: Inhaled Nitric oxide
IV: Interventricular
LV: Left ventricle
MRI: Magnetic resonance imaging
NICU: Neonatal Intensive Care Unit
NIRS: Near-Infrared Spectroscopy
PGE1: Prostaglandins
PH: Pulmonary hypertension
PRAM: Pressure Recording Analytical Method
PVR: Pulmonary vascular resistance
rSO2c: Regional cerebral tissue oxygen saturation
rSO2s: Regional splanchnic tissue oxygen saturation
RV: Right ventricle
SVR: Systemic vascular resistance
TAPVR: Total anomalous pulmonary venous return
VGAM: Vein of Galen malformation
VSD: Ventricular septal defect
