# Supplementary material for: Use of levosimendan in hemodynamic management of heart failure in two neonates with intracranial arteriovenous shunts: a case series
Source: Ital J Pediatr. 2023 Oct 15;49:141. doi: 10.1186/s13052-023-01537-1 (PMC10577993; doi:10.1186/s13052-023-01537-1)
Supplement: Supplementary file 1 — Supplementary Material 1 [file 13052_2023_1537_MOESM1_ESM.docx]

**ITJP-D-23-00196
Use of levosimendan in hemodynamic management of heart failure in two neonates with intracranial arteriovenous shunts
Francesca Landolfo; Paola Giliberti; Domenico Umberto De Rose; Flaminia Pugnaloni; Alessandra Santisi; Claudia Columbo; Ludovica Martini; Maria Paola Ronchetti; Luca Di Chiara; Alessandra Toscano; Carlo Gandolfo; Andrea Dotta; Irma Capolupo
Italian Journal of Pediatrics**

Reviewer reports:

Reviewer #1:

I have read the revised version of the paper, and I'd like to thank the Authors for the good job.

**Authors: Thank you for your appreciation of the revised version of the paper.**

I am convinced of the request made regarding the need to present the data of the two patients in a different form with respect to the statistical comparison carried out. I understand the Authors' impression of conveying to the reader the significant difference in hemodynamic parameters after treatment with levosimendan. Unfortunately, in my opinion, this is only an impression as it is methodologically incorrect. The test used has no meaning in this case. I therefore ask the authors to review this decision, or I ask the Editor to have a statistician evaluate this position.

**Authors: We removed the t-student and p-values, as requested. We left only the values in the tables.**

**We also improved the paper with a new figure showing the trend of cardiac index and oxygen delivery index in the two patients.**

Reviewer #2:

The overall quality of the modified version of the manuscript has been improved, and, according to my opinion, now the paper is suitable for publication in IJP.

**Authors: Thank you for your appreciation of the revised version of the paper.**
